# A Review of the General Principles of Physiology

**Published:** 1822-12-01

**Authors:** 


### VIII.

*A Review of some of the general Principles of Physiology, with the Practical Results to which they have led.* By A. P. W. Philip, M. D. F. R. S. Edinb.

Dr. Philip has laid before the public a paper with the above title, in the three last numbers of the Journal of Science edited at the Royal Institution. As it contains a retrospection of the various physiological investigations of its author, we shall take this opportunity of laying before our readers an enumeration of the physiological points ascertained by his experiments. From the nature of the paper, however, and the condensed style in which it is written, it is impossible to follow the usual plan of analysis in giving an account of it. We shall therefore lay before our readers only a statement of the facts with the general inferences to which the author has been led, and these we shall generally give in his own words. In this way we think we can best convey to the reader a distinct and correct view of the contents. Before proceeding farther, it is necessary to call the reader's attention to the sense in which Dr. Philip uses the term vital principle, the vague signification of which has led to much inaccurate reasoning. In the commencement of his paper he defines it in the following manner, and afterwards particularly requests that his definition of it may be kept in mind.

"Both the animal and vegetable world differ from inanimate matter, in affording a peculiar class of results when impressed by other agents, whether chemical or mechanical. The quality on which the peculiarity of these results depends, has been termed the vital principle. Whether this principle be something superadded to bodies, or only a peculiar arrangement of their constituent parts, we have no means of ascertaining. The fact is, that it bestows on them certain properties. It is essential that its name should convey this fact, and no more." 97.

It would appear from the experiments of Haller, that the heart is wholly independent of the nervous system; why, then, it was justly asked, is it supplied with nerves, and influenced by the passions? The first physiologists of Europe, Fontana, Procsaska, Soemmering, Bekrends, Earnest Platner, Winslow, Winkle, Johnson, Unzer, Levat, Peffinger, Scarpa, Bichat, Le Gallois, and others, have in vain endeavoured to reconcile these apparent contradictions. The committee of the Institute of France conclude their report on the experiments of Le Gallois in the following manner:—

"The foregoing, the report continues, is a short but faithful account of the principal systems by means of which authors have, since the discovery of the circulation of the blood to this day, attempted to explain the motions of the heart. On taking a general view of those invented before Haller, we remark, that in all of them the nervous power is considered in one way or other as one of the conditions essential to the production of the motions of the heart, and it is always and only in the brain that they place the seat of it. The cardiac nerves therefore had a determined use in all these systems, and one could easily understand why the heart is subject to the empire of the passions: but it was impossible to explain why the circulation continues in acephalous animals, and why in experiments on animals the interruption of all communication between the brain and the heart does not stop the motion of the latter. Since the time of Haller, irritability has been the basis of all these systems. In regarding that property as essential to the fibre, and independent of the nervous influence, the circulation in acephalous animals and the different phænomena observed in the experiments alluded to, present nothing that is not easily understood; but the use of the nerves of the heart, and the influence of the passions on that organ become inexplicable. The necessity of removing these difficulties has produced two parties among the supporters of irritability. The one, zealous favourers of the doctrine of pure irritability, called to their aid the most improbable hypotheses, and all their efforts have only served to prove how difficult it is to support the cause they espouse. The other confounded the nervous power with irritability, which they consider as one of the functions of that power; but they have been obliged to admit, either with respect to the seat or the mode of existence of the nervous power, conditions, which by their own confession are far from being demonstrated, respecting which they are not agreed, and which in the application they make of them to the motions of the heart, either do not wholly remove the old difficulties, or create new ones." 105.

M. Le Gallois imagined, that he had removed the difficulty by proving that the power of the heart is wholly independent of the brain, and derived from the spinal marrow; and the Committee of the Institute, after seeing his experiments repeated, admitted his conclusions, and published a long memoir in support of them; declaring them to be the most important which had been promulgated since the time of Haller.

Dr. Philip has pointed out the insufficiency of the grounds on which M. Le Gallois and the Committeee founded their conclusions, and has demonstrated, by experiment, that the power of the heart is as independent of the spinal marrow as of the brain. He has, also, it is generally admitted, removed the difficulties in question, by showing, by direct experiment, that although the powers both of the heart and bloodvessels, are equally independent of every part of the nervous system, they are every where subjected to the influence both of the brain and spinal marrow. He has, also, demonstrated, by the 32d Experiment in his Inquiry into the Laws of the Vital Functions, that the power of the muscles resides in themselves, and, consequently, that any nervous influence remaining in them after the division of their nerves, is not the cause of their remaining excitability, as the opponents of Haller advanced in opposition to him. Dr. Philip adds,—

"The foregoing facts afford an easy solution of the difficulties stated in the report of the Committee of the Royal Academy of Sciences. The heart continues to act for some time after it is removed from the body, and performs its functions in the fœtal state, when no brain, and, as the committee ought to have added, no spinal marrow has existed, because it has no direct dependence on any part of the nervous system. The heart is supplied with nerves, and subject to the influence of the passions, because, although independent of this system, it is capable of being influenced through it." 109.

By the whole of Dr. Philip's experiments on this subject, he has pointed out that the difference in the nature of the muscles of voluntary, and those of involuntary, motion, is not, as Haller maintained, that the one is subjected to the command of the nervous system, while the other is placed beyond it; but that, both being subjected to its influence, this difference consists in its affording the sole stimulus of the former, but only an occasional stimulus to the latter class of muscles. Dr. Philip observes,—

"Much has been said of the cause of the one set of muscles being subjected to the will, while the other is independent of it; but if the mind be freed from preconceptions, we can surely be at no loss to account for this difference, when we know that the muscles of involuntary motion are all exposed to the constant, or constantly renewed action of stimuli over which the will has no power; while the sole stimulus of the muscles of voluntary motion is wholly subjected to it. Besides, the action of the former muscles produces no sensible effect. We will to move a limb, not to excite a muscle; we wish to handle, for example, and on trial find that we can move the fingers, but there is no act of volition which could be performed through the medium of the heart and blood-vessels. If we had no wish to handle, the muscles of the fingers, of course, could never have become subject to the will. Few have any command over those of the external ear; and it deserves to be remarked, that the will influences the rectum and bladder, the only internal organs which can assist in accomplishing an end desired. It seems unnecessary to add, after what has been said, that the ganglions by no means intercept the influence of the brain and spinal marrow in its course to the muscles of involuntary motion, as some have supposed." 110.

Dr. Philip prosecuted his inquiries into the relation between the muscular and nervous systems, much farther than Haller attempted to go, and has, by various experiments, ascertained that the nervous influence is supplied to these different classes of muscles from different sources. To the muscles of voluntary motion, it is supplied from the several parts of the brain and spinal marrow from which their nerves originate, while to the muscles of involuntary motion, he has proved by an extensive set of experiments, it emanates from every part of the brain and spinal marrow, through the interposition of the ganglions, which are thus shown to be the means of concentrating the influence of every part of these organs, to bestow it on all those parts which participate the influence of the ganglionic nerves. The use of the ganglions is thus pointed out, which could not, of course, have been conjectured till it was demonstrated, that the parts supplied by ganglionic nerves, share the influence of every part of the brain and spinal marrow. Dr. Philip remarks,—

" We may easily conceive why the muscles of voluntary motion are excited when those parts of the brain or spinal marrow from which they receive their nerves are stimulated; but it seems at first view more difficult to account for the heart and other muscles of involuntary motion being subject to the influence of every part of these organs. We cannot suppose that they receive nerves from every part of them. We know, indeed, that no organ does so. The following seems to be the state of the question. We see some parts influenced by every part of the brain and spinal marrow; others only by small parts of them. In the latter instances, we see directly proceeding from those small parts the nerves of the part influenced. In the former instance, namely, where the part is influenced by all parts of the brain and spinal marrow, we do not see nerves going directly from all parts of these organs to the part influenced, but we see this part receiving nerves from a chain of ganglions to which nerves from all parts of them are sent. It is, therefore, evident, from direct experiments, that the nerves issuing from ganglions convey to the parts, to which they send nerves, the influence of all the nerves which are received by these bodies." 266.

Dr. Philip then ascertained, by means of experiment, on which alone he has founded all his inferences, that the influence of every part of the brain and spinal marrow is thus combined for the purpose of effecting the secreting, and other assimilating processes, which, he has shown, are deranged, if the influence of any considerable part either of the brain or spinal marrow be withdrawn, while, at the same time, he pointed out, that all the parts supplied with ganglionic nerves are, more or less directly subservient to these processes. Dr. Philip continues,—

"Thus, we perceive, the necessity of every part of the function which the ganglions appear to perform. A combination of the whole nervous influence is necessary to the due formation of the secreted fluids: and that there may be, under all circumstances, both a due supply of the fluids to be acted upon, and a due removal of those prepared, whether for the functions of life, or for the purpose of being thrown out of the system, it is necessary, as appears from what has just been said, that the powers which convey all these fluids should be subjected to the influence by which secretion is performed. This function, it is evident, requires a more regular supply of fluids than could have been obtained, had the usual action of the vessels depended on the nervous system, which is subject to continual variation; but had not this system been capable of influencing the vessels, not only no change in it could have influenced the flow of secreted fluids, but every occasional increase of the influence of the nervous system, supplied to secreting surfaces, finding no increase of fluids to act upon, would necessarily have excited disease. Thus, it is requisite that the power of the sanguiferous should be independent of the nervous system, yet capable of being influenced by it; as from direct experiment, we have just seen, it is found to be." 274.

By the experiments on this subject brought into comparison with others, he has shewn that, the processes of secretion and assimilation, as well as that process by which animal temperature is supported, are the results of the action of the influence supplied by the nervous system, on the circulating fluids. He remarks,—

"Here a question of great importance in the animal economy arises. As it appears, from the experiments just referred to, that the nervous power is equally essential with the circulation of the blood, for maintaining the functions of secretion and assimilation, what are the parts they severally perform in these functions? It is evident, that the extreme parts of the sanguiferous and nervous systems are connected in a way very different from that in which these systems are connected in other parts. The heart and vessels of circulation, we have seen, can perform their function after the influence of the nervous system is withdrawn. The function of the secreting vessels immediately ceases on the interruption of this influence. We must suppose, therefore, either that the influence of the nervous system bestows on the extreme vessels the power of separating and re-combining the elementary parts of the blood, or that the vessels only convey the fluids to be operated upon by this influence.

" Experiments, to which I have already referred, prove that the most minute vessels which can be seen by a powerful microscope in the web of a frog's foot, are independent of the nervous system. The motion of the blood is as rapid, and the circulation in the foot presents precisely the same appearance after as before the slow destruction of the brain and spinal marrow. If the power of the vessels of secretion had been lost by the interruption of the influence of the nervous system, would not this have necessarily occasioned some change in the distribution and motion of the blood in the web? The conclusion from these experiments is strengthened by others. In those in which the secreting power was destroyed either by the division of the eighth pair of nerves or the destruction of part of the spinal marrow, there did not necessarily appear to be a defective supply of fluids. In the stomach they were often as copious, sometimes more copious, than usual; and the latter was almost always the case in the lungs. The fault seemed to be, that a due change on them had not been effected. We know that the vessels of circulation possess no powers but the elastic and the muscular, or what in many of its properties resembles the latter. Can we suppose, that the vessels of secretion, which are only a continuation of those of circulation, all at once assume a different nature; or is it at all consistent with our knowledge of the phenomena of chemistry to suppose, that by any influence the powers just mentioned, or indeed any that can be supposed to belong to vessels, could be enabled to separate and re-combine the elementary parts of the blood? The first of the above positions being set aside, it seems a necessary inference from the experiments referred to, that in the function of secretion, the vessels only convey the fluids to be operated upon by the influence of the nervous system." 272.

With regard to the temperature of the animal body, Dr. Philip makes the following observations,—

" It appears, from experiments above referred to, that the destruction of any considerable portion of the spinal marrow, deranges the function of secreting surfaces. Together with this effect, it was always found to lessen the temperature of the animal, more or less, according to the extent and importance of the part destroyed.* Some years previously, Mr. Brodie, in the Croonian Lecture for 1810, gave an account of experiments which led to the inference, that the maintenance of animal temperature is under the influence of the ner-

---

* *Exper. Inquiry*, p. 161, *et seq.* second edition.

vous system, and in the Philosophical Transactions of 1812, he relates additional experiments, tending to strengthen this inference. The experiments related in the inquiry just referred to, seem in a striking manner to confirm the opinion of Mr. Brodie. He found that poisons impairing the vigour of the nervous system, impair the temperature. It appears from my experiments, that lessening the extent of this system, by destroying part of the spinal marrow, has the same effect.

"Thus, it follows, that the temperature of the animal body depends on the state of the nervous system; but many observations point out that it depends also on that of the powers of circulation. When the power of the heart and vessels is greatly impaired, so that the motion of the blood languishes, the temperature falls. If by exercise, or the use of stimulants, we increase the action of the heart and vessels, the temperature in the same proportion rises. When there is a natural defect in the organs of circulation, and particularly when this defect is such as prevents the blood passing through the lungs, with the freedom necessary to its healthy state, the temperature is found below the natural standard. The reader may consult a paper on this subject, by Mr. Earle, in the seventh volume of the Transactions of the Medico-Chirurgical Society, in which there are many excellent observations. As we proceed, we shall find proofs founded on direct experiments, that the temperature depends on the state of the circulation, and particularly on the passage of the blood through the lungs, which to detail here, would too much anticipate some of the other parts of the subject.

"Whether caloric be a substance, or as some of the first chemists of our time are inclined to believe, only a certain motion of the particles of bodies, it is of course foreign to this paper to inquire; but it appears from the foregoing observations, and will, I think, appear still more strikingly from those I shall have occasion to add, that the maintenance of animal temperature must be ranked among the results of the action of the nervous system on the blood. It is on this account that I have elsewhere said, *that if caloric be regarded as a substance*, its evolution in the animal body must be ranked with the secreting processes; the definition of secretion, I conceive, being the evolution of a *tertium quid*, in consequence of that action." 275.

Dr. Philip maintains, that nerves are only capable of conveying impressions to and from the brain or spinal marrow, and, consequently, that these organs are concerned in all cases of sympathy; there being no evidence that impressions are ever communicated from one nerve to another, independently of the intervention of one of them, "a position," he adds, "farther illustrated by the able investigations of Mr. Charles Bell, which have afforded new and important views of the distributions and uses of certain nerves."

Dr. Philip has devoted many experiments to the ascertaining the line of distinction between the nervous and sensorial systems, which have hitherto been confounded, and has shown, experimentally, that, as the muscular power can exist after the influence of the nervous system is withdrawn, this influence can exist, and is capable of all its functions, after the sensorial power is withdrawn.

It appears, from his experiments on this part of the subject, that the muscular, nervous, and sensorial powers have no direct dependence on each other; yet are all so connected in the more perfect animals as to be incapable of long surviving each other. He has pointed out that it is through the medium of respiration that the muscular and nervous depend on the sensorial power, this being the only vital function to which the sensorial power is necessary, and the first which fails in dying in consequence of the sensorial power being the first which is withdrawn. Dr. Philip remarks,—

"M. le Gallois finds a great difficulty in explaining why respiration should cease on the removal of the brain.

"'Il est donc certain que la vie de tronc n'a son principe immédiat ni dans le cerveau, ni dans aucun des viscères de la poitrine et de l'abdomen; mais il ne l'est pas moins, que tous ces viscères sont indispensables à son entretien. Or, en considérant sous quel rapport ils le sont, les faits énoncés plus haut prouvent évidemment que, quant au cerveau, les phénomènes mécaniques de la réspiration, c'est-à-dire, les mouvemens par lesquels l'animal fait entrer l'air dans ses poumons, dépendent immédiatement de ce viscère. Ainsi c'est principalement en tant que l'entretien de la vie dépend de la réspiration qu'il dépend du cerveau; ce qui donne lieu à une grande difficulte. Les nerfs diaphragmatiques, et tous les autres nerfs des muscles qui serve aux phénomènes mécaniques de la réspiration, prennent naissance dans la moëlle épinière, de la même manière que ceux de tous les autres muscles de tronc. Comment se fait-il donc qu'après le décapitation, les seuls mouvemens inspiratoires soient anéantis, et que les autres subsistent? C'est la à mon sens, un des grands mystères de la puissance nerveuse; mystère qui sera dévoilé tôt ou tard, et donc la découverte jettera la plus vive lumière sur la mécanisme des fonctions de cette merveilleuse puissance.'

"This difficulty appears to me to arise from M. le Gallois's having regarded respiration as a function wholly dependent on a combination of the nervous and muscular powers; whereas it seems evident, I think, that the sensorial power also shares in it. The muscles of respiration are, in the strictest sense, muscles of voluntary motion; we can at pleasure interrupt, renew, accelerate, or retard their action; and if we cannot wholly prevent it, it is for the same reason that we cannot prevent the action of the muscles of the arm, when fire is applied to the fingers. The pain, occasioned by the interruption of a supply of air to the lungs, is greater than can be voluntarily borne. Respiration continues in sleep for the same reason that we turn ourselves in sleep when our posture becomes uneasy. It continues in apoplexy for the same reason that the patient generally moves his limbs if they are violently irritated. If respiration continues in apoplexy when no irritation of the limbs, however violent, excites the patient to move them, it arises from the interruption of a supply of air to the lungs, producing a greater degree of irritation than we are able to produce by other means. As the insensibility increases in apoplexy, the breathing becomes less frequent; and when the former becomes such that no means can longer excite any degree of feeling, the breathing ceases.

"By a certain sensation a desire is excited to expand the chest. This is an act of the sensorium. Till this act take place, both the nervous and muscular powers, by which its expansion is effected, are inert; it is in vain that these powers exist, if the power which calls them into action be lost. Thus the removal of the brain puts a stop to respiration." 98.

While Dr. Philip maintains that it is impossible to refer the sensorial functions to any more general principle, he has, by experiments, the accuracy of which had long been questioned, but is now universally admitted, traced the nervous functions to the agency of galvanism.

He has shewn, that the whole of these functions may be performed by this mean, after the nervous influence has ceased to operate, provided the parts still retain their vitality; and has, on the other hand, proved, that that influence can be made to leave the nerves, and pass through conductors of galvanism without being rendered incapable of its functions. Thus, in both ways establishing the identity of these agents.

Lastly, he has pointed out, what is perhaps of greater importance than any other fact in estimating the nature of the animal functions, that it is a necessary inference from his various experiments, that the sensorial functions alone are the results of vital parts affecting each other by their vital properties; all the other vital functions being the results of inanimate agents acting on vital parts. To prove by experiment, indeed, that the nervous influence can perform its functions after it has been made to leave the nerve and pass through other conductors, seems alone sufficient to evince that it is an inanimate agent. Hence it is that we can, by artificial means, imitate the nervous functions, that these bear so strong an analogy to the phenomena of inanimate nature, and that we can refer them to a more general law, that is, class them with other phenomena which arise from the same power, whose effects, as Dr. Philip expresses it, are modified but not wholly changed by one of the agents in these functions being possessed of vitality; while the sensorial functions, on the other hand, bear no analogy to any other phenomena, and can be referred to no more general principle, vital parts affecting each other by their vital properties only in the animal body; for all the functions of the vegetable world, according to the principles which result from Dr. Philip's experiments, arise from the action of inanimate agents on vital parts. Dr. Philip observes—

"As the properties of the vital principle do not differ from those of inanimate matter merely in degree or by any other modification, but have nothing in common with them, it follows, that when parts endowed with this principle affect each other only by their vital properties, the result must be such as bears no analogy to any of the properties of inanimate matter; and, consequently, that in all processes which have any such analogy, one of the agents must operate by the properties of this matter. We have seen that the characteristic difference in the sensorial and nervous functions is, that the former bear no analogy, the latter a very striking one, to those properties. On the other hand, we see the organs of the nervous system impressed by external objects, those of the sensorial system only through other vital parts. The nervous system is evidently the connecting link between the sensorium and the world which surrounds us. It consists of parts endowed with the vital principle, yet capable of acting in concert with inanimate matter; receiving impressions from it, and if the position just stated be correct, capable of impressing it; for there can be no stronger analogy than that which subsists between the secreting processes effected by the influence of the nervous system, and the chemical processes which take place in such matter; and if an inanimate agent be employed in these processses, its supply and application must be regulated by the vital powers of the nervous system. Whether this agent be a distinct being, or only a peculiar modification of the particles of bodies, is not the question; all the essential inferences are, in either case, the same. The phenomena of electric animals are here in point. We see their nervous system collecting and applying, even according to the dictates of the will, an inanimate agent." 104.

We shall finish our view of the physiological part of the paper, which we have taken great pains to render connected and satisfactory, by quoting from it the following short recapitulation.

"It appears from all that has now been laid before the reader that there are three distinct powers in the animal system which have no direct dependence on each other, for we have seen the muscular surviving both the sensorial and nervous power, and the nervous the sensorial and muscular power; and nobody has supposed that the sensorial power has any dependence on either of the others, except as far as they are necessary for the maintenance of its organs, in which respect the nervous and muscular in the more perfect animal are equally, though not so immediately, dependent on the sensorial power.

"The nervous and muscular powers are, on the one hand, the direct means of maintaining the life of the animal, and on the other, of connecting it with the external world; the former receiving impressions from that world, the latter communicating impressions to it. All the functions of both powers bear a strong analogy to the properties of the world with which they are thus associated; we therefore have reason, according to principles above stated, to believe that all these functions, as is evidently the case with many of them, are the results of inanimate agents acting on vital parts.* There is none of them, as appears from the experiments which have been referred to, which may not be excited by artificial means as long as its organs retain the vital principle; and it is a remarkable fact, that they are all capable of being excited by one agent, and that an agent universally diffused, which we know from other facts to be intimately connected with the animal economy, and which in some of its most characteristic properties the influence of the nervous system resembles. Lastly, we know that an agent of the same nature with that of which we now speak, electricity, is in some animals capable of being collected and applied by the organs of the nervous system.

"When from the nervous and muscular we turn to the sensorial functions, we perceive results which have lost all analogy to those of inanimate matter. They have only an indirect effect in maintaining animal life, and are excited by no impressions but those communicated through the nervous system, and consequently are the results of living parts acting on each other. Hence it is, that they are the first functions which cease when the vital power begins to fail. In the nervous and muscular functions an inanimate agent excites the languid powers of life. In the sensorial functions, the functional power and the stimulus which excites it, being equally vital powers, fail together.

"When the nature of the sensorial functions is kept in view, we cannot be surprized that the attempts to refer them to a more general principle have proved so futile. To what other principle shall we refer the effects of the vital parts of animals on each other, when it is in animals alone that such parts ever influence each other? Even in vegetable life we find nothing analogous to the sensorial functions. All its processes bear the same analogy to the properties of inanimate matter, which we observe in the functions of the nervous and muscular systems of animals, and are, therefore, the results of inanimate agents acting on living matter.† Much less can we

---

* "The nervous power, by which the impressions on the organs of sense are conveyed to the sensorium, receives those impressions from inanimate matter. Even the heart is excited by inanimate agents, for although the blood be alive, it is by its chemical properties and bulk that it excites the heart and vessels, as appears from rendering the blood more or less stimulating, and greater or less in quantity."

"† It is here worthy of remark, that many phenomena render it probable that there is a continual passage of the electric influence through plants, to which both their form and position are peculiarly adapted."

look for any analogies of this kind in inanimate nature itself. Such reveries may please as the creations of the poet, but admit not of serious discussion. We are charmed with the flights of Lucretius, but we see only the perversion of philosophy in the reasonings of Hartley." 109.

In the foregoing review we have shortly enumerated the discoveries for which we are indebted to Dr. Philip. The public is left to compare them, without partiality or prejudice, with those of former or present physiologists; and to assign to Dr. Philip the place which belongs to him. He has attempted to raise no superstructure which has not experiment for its basis; and seems to have had it in view to draw a correct line between what is and what is not known. He has pointed out the vanity of enquiring into the nature of the vital principle and sensorial power, and shewn that the study of them must be confined to the observation and arrangement of their phenomena. In addition to what he has done on subjects which had long been under discussion among physiologists, he has carried his investigations into the more complicated functions of the nervous system, which were very generally supposed to be placed beyond the reach of experiment, and has given an unanswerable proof of his success by exhibiting these functions performed by artificial means.

It is a valuable, and, it will be admitted, an unusual feature in the physiological investigations of Dr. Philip, and a striking guarantee of their accuracy, that they have led immediately to improvements in the practice of medicine. We shall quote the whole of what is said in the present paper, relating to their practical results. The importance of these results will be a sufficient apology for the length of the quotation.

"If the foregoing inferences from the various experiments which have been referred to be correct, it is reasonable to suppose that they may be beneficially applied to the practice of medicine. The view of the different functions of the animal body, and their mutual dependence on each other afforded by those experiments, cannot, in that case, fail to be of use in explaining the nature and regulating the treatment of the deviations of these functions from the healthy state, particularly in the diseases whose symptoms are most influenced by the mutual sympathy of the vital organs.

"In a Treatise on Indigestion, I have attempted its application to an extensive class of these diseases. But I here wish chiefly to direct the reader's attention to the practical results from the experiments which relate to the influence of galvanism on the animal body.

"They led me more than six years ago to the employment of this agent in diseases, which seem to arise from a defect of nervous power, particularly habitual asthma and indigestion; and an account of its effects in those diseases was published in the *Philosophical Transactions* of 1817. It is now admitted, I believe, by all who have witnessed them, that in the former disease, and under certain circumstances of the latter, galvanism is the most effectual means of relief which we possess.

" In its employment, we must constantly guard against the inflammatory diathesis, both because it tends to produce this diathesis, and because the diseases to which it is adapted, for reasons pointed out at length in the Treatise on Indigestion, to which I have just referred, have the same tendency. As any considerable degree of the inflammatory diathesis not only obviates the beneficial effects of galvanism, but renders it injurious, the constant superintendence of a well-informed practitioner is necessary. I need not here enter more particularly into this part of the subject, which has been done in that treatise, and in my *Experimental Inquiry into the Laws of the Vital Functions*, in which the reader will find a detail of cases cured, or relieved by galvanism. To its effects in one case of considerable importance I shall beg leave more particularly to direct the reader's attention, because it is only since I last had occasion to mention the subject publicly, that I have witnessed them. Mr. Earle some time ago asked me, if I thought galvanism a probable means of relief in dyspnœa and indigestion, arising from disease of the spinal marrow. I did not hesitate to recommend a cautious trial of it, referring Mr. Earle to what I had said of such cases in the last part of the above-mentioned Inquiry. I am happy to say the result has fully answered our expectations, as appears from the following letter which Mr. Earle did me the favour to address me.

*George Street, August* 14, 1822.

" My dear Sir,

" I have much pleasure in transmitting to you the following account of the trials made with galvanism at St. Bartholomew's Hospital. The first case is that in which you witnessed its first application.

" Elizabeth Pepperall, aged 17, of fair complexion, and light hair, was admitted into St. Bartholomew's Hospital in August 1821, in consequence of an affection of the spine, which had existed for about a year and a half. At the time of her admission, it appeared, that almost all the dorsal and lumbar vertebræ were affected. She had nearly lost all power over her lower extremities and pelvic viscera; and she complained of very severe cramps at the pit of the stomach, and acute pain in the course of the costal nerves, which was much increased by pressure on the ribs, or any attempt at a deep inspiration. Her general health was much deranged; her pulse was very rapid, with occasionally severe palpitation of the heart, and constant dyspnœa. Her digestive powers were greatly impaired, she had no appetite; and could only digest a small portion of stale bread and some milk and water. Even this meal was always followed by uneasy sansations at her stomach, and an increase of head-ach, from which she was hardly ever free. Her bowels were obstinately costive, and the urine was scanty, and deposited large quantities of lithate of ammonia.

"She was placed on one of my invalid beds, which enabled her to remain in a state of uninterrupted rest; and after the repeated application of leeches, issues were made on either side of the dorsal spine, and subsequently in the lumbar region. The issues were kept actively open, and the strictest attention was paid to her general health. The spine very gradually became less sensible, and the power over the pelvic viscera and lower extremities slowly returned; still, however, her stomach was incapable of digesting any other food than bread and milk and water, her head-ach remained nearly unabated, and her breathing was habitually difficult. She was in this state when you saw her, and the galvanism was first administered (December 19.)

"'A trough containing plates of about three inches was employed. The positive wire was applied to the nape of the neck, the negative a little below the pit of the stomach. No sensation was at first produced by twenty plates; but after the sensation was excited, she could not endure more than twelve. The first sensation she experienced caused her to take involuntarily a sudden and deep inspiration. The galvanism was applied for about a quarter of an hour, at the end of which time, her breathing became much freer than it had been for many months. Of this she repeatedly expressed herself perfectly certain, at the same time she felt considerable uneasiness at the stomach. She was slightly hysterical, in consequence of the agitation she had experienced, but her breathing was tranquil during the whole evening.

"'With a view to remove the tenderness in the epigastrium, leeches were applied to the region of the stomach, and the whole plan of treatment adapted to the secondary stage of dyspepsia was resorted to. When the tenderness had somewhat abated, the galvanism was repeated with more decided relief to the breathing, and without causing much uneasiness at the stomach.

"'After several applications of it, the relief she experienced in her breathing lasted for two or three days, and at length it was only necessary to repeat it occasionally. The effect of its administration was uniformly the same; a most sensible and speedy relief from a state of anxious breathing to perfect ease and repose. Its beneficial effects were not, however, confined to the respiration; the powers of her stomach greatly improved, and she was able to digest a small quantity of meat or the yolk of an egg without pain. As her stomach improved, she lost the distressing head-ach, which had so constantly attended, as at one time to lead me to apprehend the existence of disease in the brain, having met with other cases in which scrofulous affection had existed in the brain and spine at the same time. Her progress from this time was uniform, and far more rapid than it had been before; and in about two months, the catamenia, which had been suspended from the commencement of the disease, returned.

" 'The patient was sufficiently recovered to leave the hospital, and return to her friends at Dartmouth early in July; at which time she was able to walk with very little assistance, and without experiencing the least pain in her back. On reviewing the circumstances of this case, I have not the least hesitation in stating my decided opinion of the great benefit which was derived from the employment of galvanism, not only in affording temporary relief to the breathing, but in improving the secretions, and thus materially contributing to the ultimate recovery of the patient. I feel particularly happy that the patient was in a public hospital, and that the means were employed in the presence of many intelligent medical friends and pupils, who were all equally satisfied with myself of the essential and permanent benefit which she derived from the administration of galvanism.

" 'It was employed in two other similar cases in the same hospital, those of Ann Baillies, and Maria May, in which it produced similar good effects, except that in one of these, the improvement of the general health, although not less than in the other cases, did not appear to have the same beneficial effect on the disease of the spine. It was tried in another case of spine disease, which was attended with fits of spasmodic asthma. These, as I was taught to expect, from the observations you have published on this subject, it failed to relieve. It is remarkable, that in the case of Ann Baillies, in which the pulse was from 140 to 150, and very weak, the use of the galvanism always rendered it stronger, and brought it down from thirty to forty beats in the minute.

" 'From observing the good effects of galvanism on the secretions of the stomach, I was induced to make a trial of it in a case of deafness, accompanied with a total want of secretion of cerumen in the right ear. Its first application produced a watery secretion, which by perseverance gradually assumed the taste and all the other characters of cerumen. The hearing was greatly improved in both ears, but how far this was to be ascribed to the restoration of the secretion is rendered doubtful, in consequence of a tumour having at the same time been removed from the tympanum of the left ear by the repeated application of caustic.

" 'The foregoing facts you are perfectly welcome to make any use of, should you think them deserving of notice, and I am,

My dear Sir,

Very sincerely, yours,

HENRY EARLE.'

" It appears from the foregoing statement, that in disease of the spinal marrow, galvanism is not only capable of performing the function of the diseased part of this organ, by which the vital actions are restored to a state of health, and the patient's sufferings greatly mitigated; but that, it also, as might *à priori* be expected, by thus improving the general health, indirectly contributes to the cure of the spinal disease. With regard to the last case mentioned by Mr. Earle, in which the secretion of cerumen was restored by galvanism, this, it is evident, from what has been said, can only happen when the fault consists in a defect of nervous influence, and not in a diseased state of the vessels.

"When we compare the foregoing report of Mr. Earle with the statements which I have already had occasion to make public, respecting the effects of galvanism in other diseases, may we not hope that if in so few years such has been the result of the employment of this remedy on the principles above laid down, a more extensive experience will still extend the advantages derived from it. I have repeatedly seen its use more successful than any other means in obstinate general nervous debility, in which transmission through the stomach and lungs has still appeared to me the best means of applying it. In certain species of fever, and other cases attended with deficient nervous energy, we have reason to believe that it will be found a valuable remedy.

"I may close these observations by observing, that when galvanism is not used to such extent as to occasion an inflammatory tendency, I have never seen any bad effect from it, except a sense of languor, similar to the feeling of fatigue, when its employment has been too long continued. The inflammatory tendency produced by it, according to my experience, is always easily removed; is never followed by any serious consequence; and, with a little care, may almost always be prevented. I have repeatedly observed that when the cure has advanced to a certain point, its judicious employment, so far from causing the inflammatory tendency, has, by improving the state of the secreting surfaces, relieved that caused by the disease." 115.

In this article, as our readers perceive, we have been strictly analytical, convinced that by such course we would best promote the interests of science, and the well-earned reputation of the author. In fine, we have no hesitation in expressing our opinion that the various writings, discoveries, and experiments of Dr. Wilson Philip, deservedly place him high in rank among the most illustrious physiologists, pathologists, and physicians of the age in which we live.

